# S100B Protein Stimulates Proliferation and Angiogenic Mediators Release through RAGE/pAkt/mTOR Pathway in Human Colon Adenocarcinoma Caco-2 Cells

**DOI:** 10.3390/ijms20133240

**Published:** 2019-07-01

**Authors:** Luisa Seguella, Riccardo Capuano, Mirella Pesce, Giuseppe Annunziata, Marcella Pesce, Barbara de Conno, Giovanni Sarnelli, Laura Aurino, Giuseppe Esposito

**Affiliations:** 1 Department of Physiology and Pharmacology “V. Erspamer”-Sapienza University of Rome, 00185 Rome, Italy; 2 Department of Pharmacy, University of Naples Federico II, 80131 Naples, Italy; 3 Department of Clinical Medicine and Surgery, University of Naples Federico II, 80131 Naples, Italy

**Keywords:** S100B, colorectal carcinoma, tumor microenvironment, angiogenic mediators, VEGF, RAGE, Akt/mTOR, Caco-2 cell proliferation

## Abstract

Chronic inflammation and angiogenesis are associated with colonic carcinogenesis. Enteric glia-derived S100B protein has been proposed as an “ideal bridge”, linking colonic inflammation and cancer, given its dual ability to up-regulate nuclear factor-kappaB (NF-κB) transcription via receptor for advanced glycation end products (RAGE) signaling and to sequestrate wild type pro-apoptotic wild type (*wt*)p53. However, its pro-angiogenic effects on cancer cells are still uninvestigated. To this aim, we evaluated the effect of exogenous S100B (0.05–5 µM) protein alone or in the presence of S100B blocking monoclonal antibody (mAb) (1:10^5^–1:10^4^ *v*/*v* diluted) on (1) cultured Caco-2 cells proliferation, migration and invasiveness in vitro, respectively by 3-[4,5-dimethylthiazol-2-yl]-2,5-diphenyltetrazolium bromide (MTT)-formazan, wound healing and matrigel invasion assays and (2) its effect on the release of pro-angiogenic factors, such as vascular endothelial growth factor (VEGF) by ELISA and immunofluorescence analyses. The effect of S100B alone or in the presence of S100BmAb was then investigated on RAGE/pAkt/mammalian target of rapamycin (mTOR) signaling pathway by immunoblot analysis. Our results showed that S100B markedly increases proliferation and invasiveness of Caco-2 cells, through the release of pro-angiogenic VEGF and NO paralleled to a significant decrease of *wt*p53 expression mediated by RAGE-p38 mitogen-activated protein kinase (MAPK)/pAkt-mTOR and hypoxia-inducible factor 1-alpha (HIF1α) pathways. Such effects were counteracted by S100BmAb, indicating that S100B targeting is a potential approach to inhibit colon carcinoma proliferation and angiogenesis.

## 1. Introduction

In addition to genetic, environmental and dietetic influences [1,2], chronic inflammatory conditions are well-recognized risk factors for colonic carcinogenesis [3,4]. One of the consequences of chronic colonic inflammation is neovascularization or angiogenesis, the process of formation of new blood vessels from existing ones [5] that is intimately connected with a tumor microenvironment (TME) setup enabling cancer cells to metastasize and migrate from the primitive tumor site [6]. It has been largely established that the aberrant release of different signaling molecules in the TME is able to autocrinally perpetuate the growth and proliferation of colorectal cancer (CRC) cells and their migration through the secretion and release of vascular endothelial growth factor (VEGF) [7]. By binding specific receptors, namely VEGF-R1 and 2, [8,9] VEGF stimulates neovascularization, and, in turn, cancer cell invasion and metastasis, and this negatively impacts on the prognosis of nearly 30% of early-diagnosed CRC patients [10]. The mammalian target of rapamycin (mTOR) is a protein kinase of the PI3K/Akt pathway that has been crucially involved in the activation and control of cell proliferation, survival, migration and angiogenesis. An altered mTOR signaling pathway has been described in many human cancers, including CRC [11,12]. Moreover, phosphorylation of mTOR leads to a downstream increased secretion of VEGF, leading to marked angiogenesis in both neoplastic and non-neoplastic processes [13]. Signaling molecules acting as angiogenesis promoters via the Akt/mTOR pathway or able to perpetuate colonic inflammation might thus be regarded as bridging factors, linking chronic inflammation to the carcinogenic drift. The identification of Akt/mTOR activators and their targeting, thus, may be of potential interest to develop new drugs in CRC. In this context, enteric glia-derived Ca^+2^/Zn^+2^-binding S100B protein emerges as a signaling molecule whose aberrant release plays a key role in both colonic inflammation [14] and carcinogenesis [15], given its downstream pro-inflammatory effects and mitogenic activity demonstrated in the gut, as well as in several highly malignant extra-intestinal cancers, such as melanoma and glioma [16,17,18]. At a molecular level, once over-secreted, S100B accumulates at the receptor for advanced glycation end products (RAGE) site [19], causing the downstream activation of mitogen-activated protein kinase (MAPK) with the consequent phosphorylation and activation of nuclear factor-κB (NF-κB) [20,21]. Moreover, S100B significantly inhibits the function of wild type (*wt*) p53 [22], suppressing de facto, a key physiological pro-apoptotic control of cell tumorigenesis. To date, a possible pro-angiogenic effect of S100B in colon cancer cells and its underlying molecular mechanism(s) have not been investigated yet. Given this background, the present research aimed at (1) evaluating the putative effects of S100B protein on cancer cells proliferation, invasion, migration and on the release of pro-angiogenic mediators in human colon adenocarcinoma Caco-2 cell line. We then examined S100B effects (2) on Akt/mTOR pathway and VEGF signaling in the presence or absence of specific monoclonal anti-S100B antibody, in order to confirm/exclude its specific involvement in CRC cells-related malignancy and angiogenesis.

## 2. Results

### 2.1. Effect of S100B on Caco-2 Cell Proliferation, Migration and Invasion In Vitro 

**Figure 1:**
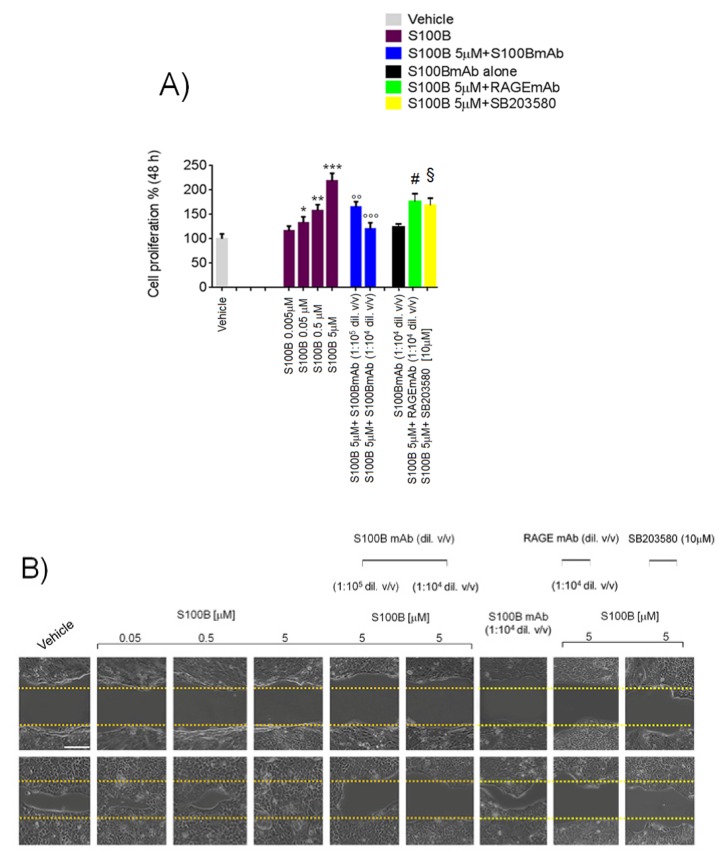

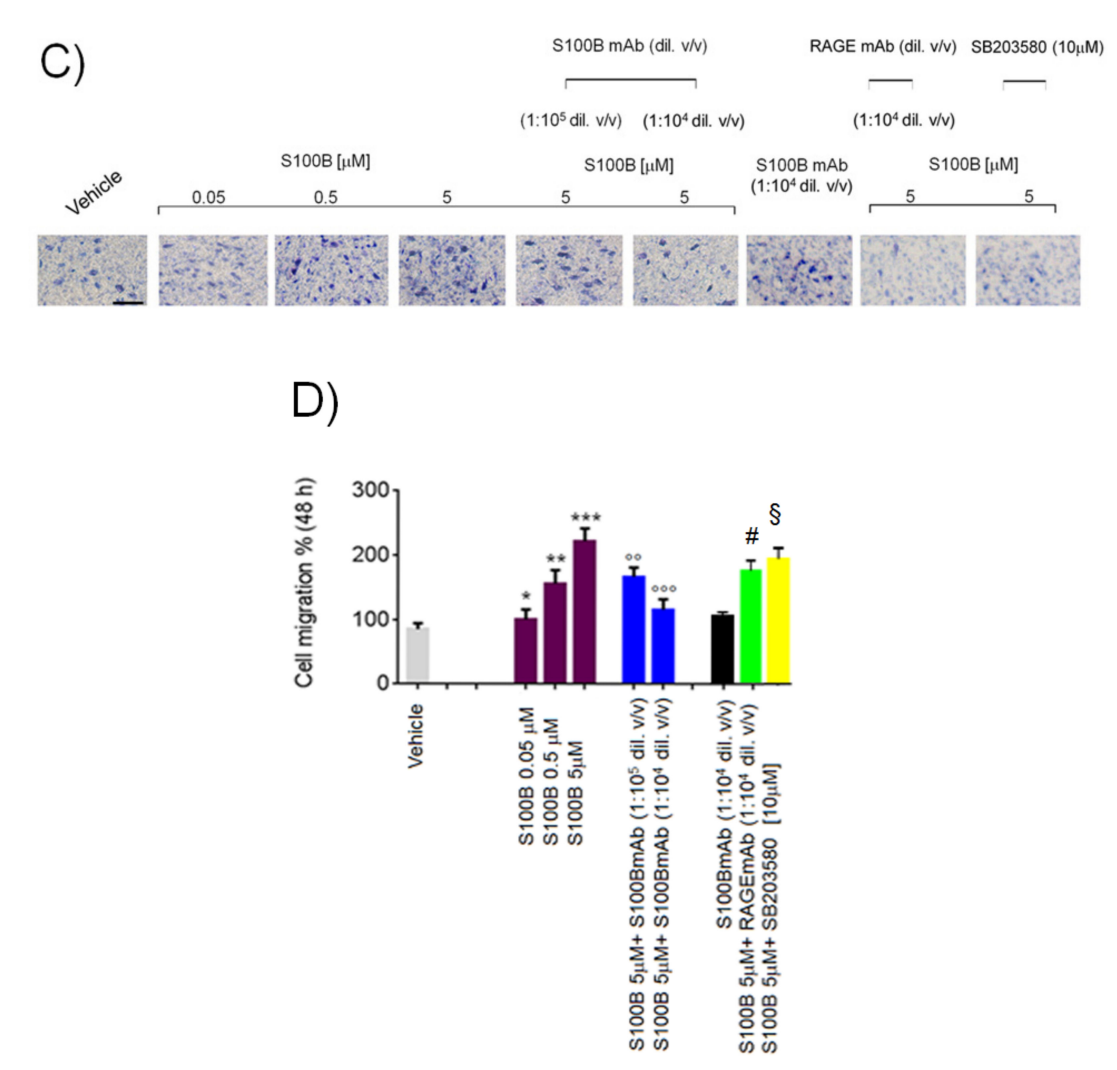

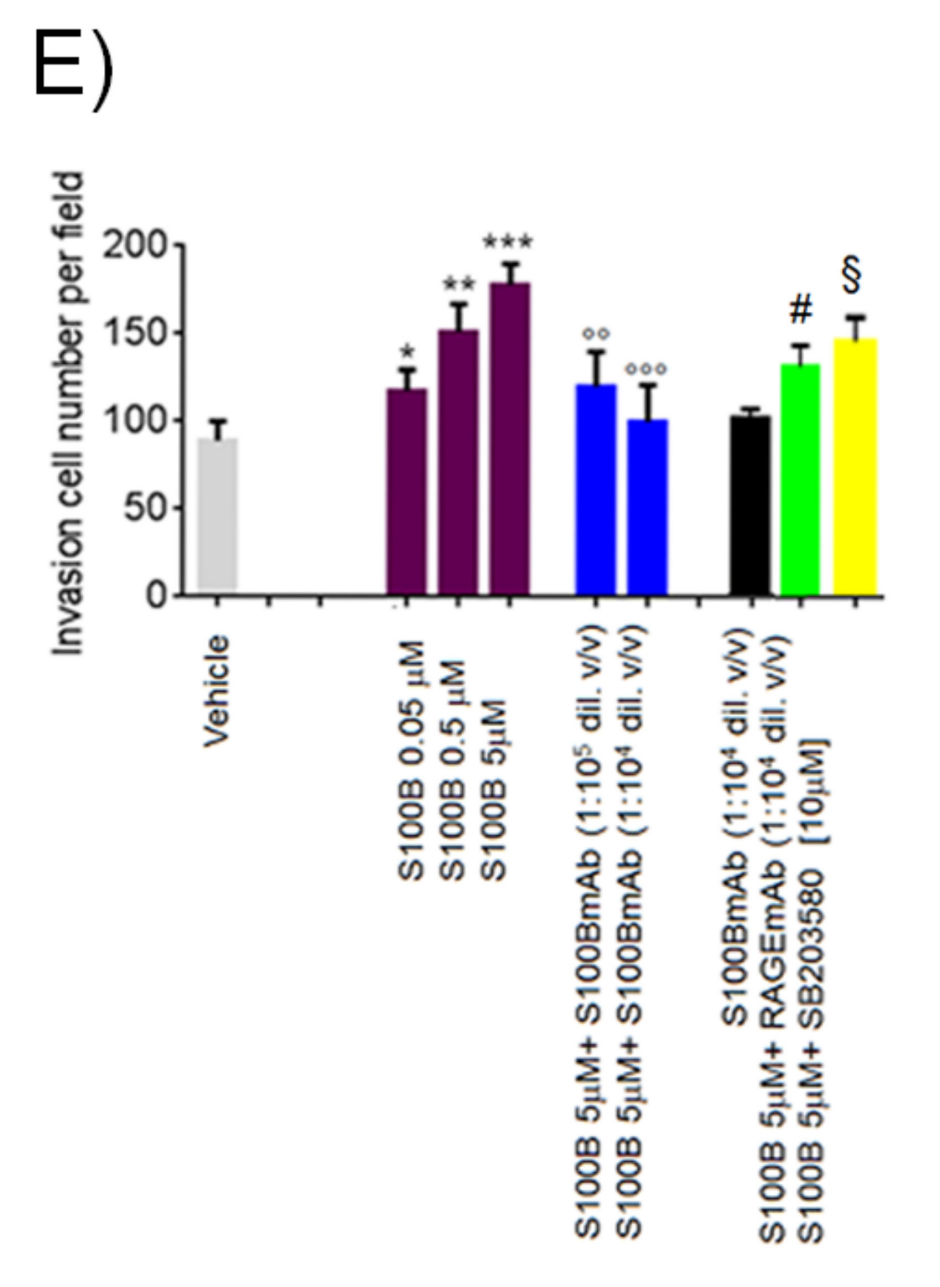
S100B stimulates Caco-2 cell proliferation, migration and invasion and its effect is blocked by S100B monoclonal antibody (mAb). (**A**) 3-[4,5-dimethylthiazol-2-yl]-2,5-diphenyltetrazolium bromide (MTT) assay showing the effect of S100B (0.005–5 µM) in the presence or absence of S100BmAb (1:10^5^–1.10^4^ *v*/*v* diluted), receptor for advanced glycation end products (RAGE)mAB (1:10^4^ *v*/*v* diluted) and/or SB203580 p38/pAkt inhibitor (10 μM) on Caco-2 cell proliferation rate at 48 h. (**B**) Wound healing assay and (**D**) the relative quantification indicating a concentration-dependent inhibitory effect of S100BmAb on cellular migration induced by S100B (0.05–5 µM). The graphs show also RAGEmAb (1:10^4^ *v*/*v* diluted) and/or SB203580 p38/pAkt inhibitor (10 μM) against S100B 5 μM stimulus at 48 h. (**C**) Cell invasion assay and (**E**) the relative quantification of invading cells following S100B (0.05–5 µM) exposure and relative inhibitory effect of S100BmAb (1:10^5^–1:10^4^ *v*/*v* diluted). Figures also show the effect of RAGEmAb (1:10^4^ *v*/*v* diluted) and SB203580 p38/pAkt inhibitor (10 μM) versus S100B 5 μM stimulus. Results were expressed as mean ± standard error (SEM) of *n* = 6 experiments performed in triplicate. * *p* < 0.05; ** *p* < 0.01 and *** *p* < 0.001 versus vehicle; °° *p* < 0.01 and °°° *p* < 0.001 versus S100B 5 μM; # *p* < 0.05 and ^§^ *p* < 0.05 respectively versus S100B 5 μM-treated cells. Scale bar: 100 µm; Magnification 10X.

Exogenous S100B (0.05–5 μM) challenge caused a significant and concentration-dependent increase of cultured Caco-2 cell line proliferation by 3-[4,5-dimethylthiazol-2-yl]-2,5-diphenyltetrazolium bromide (MTT) analysis (+16%, +33%, +58% and +111%) at 48 h in comparison with vehicle-treated (unstimulated) cells (Figure 1A). Coincubation with a monoclonal anti-S100B antibody (1:10^5^–1:10^4^ *v*/*v* diluted), concentration-dependently counteracted S100B (5 μM) effect (−22% and −43%, respectively) (Figure 1A). No significant changes were noted following S100B monoclonal antibody (mAb) (1:10^4^ *v*/*v* diluted) alone, while, both RAGEmAb (1:10^4^ *v*/*v* diluted) and p39MAPK/pAkt inhibitor SB203580 (10 μM) caused a significant reduction of cell proliferation (−20% and −23%) respectively vs. S100B 5 μM group.

The wound healing assay (Figure 1B) showed a significant increase of cell migration rate in the scratched area following incubation with S100B (0.05–5 μM), vs. the vehicle group (+25%, +84% and +161%, respectively vs. vehicle group) (Figure 1B–D) and this effect was inhibited in the presence of S100BmAb (1:10^5^–1:10^4^ *v*/*v* diluted) (Figure 1B–D), as confirmed by the observation that the distance between the borders of the wound was concentration-dependently (−25% and −48% vs. S100B 5 μM group) preserved. No significant effect in terms of cell migration inhibition was produced by S100BmAb alone (1:10^4^ *v*/*v* diluted) whereas both RAGEmAb (1:10^4^ *v*/*v* diluted) and SB203580 (10 μM), caused a marked decrease of cell migration rates (−26% and −29% respectively vs. S100B 5 μM).

In the same experimental conditions, S100B (0.05–5 μM) incubation caused a significant and concentration-dependent increase of cell invasion by matrigel assay (+27%, +70% and +100%, respectively vs. vehicle group) (Figure 1C–E). In the presence of S100mAb (1:10^5^–1:10^4^ *v*/*v* diluted) a marked decrease of cell invasion was observed (−33% and −44% respectively vs. S100B 5 μM), while S100BmAb alone (1:10^4^ *v*/*v* diluted) did not result in any significant cell invasion rate in comparison with the vehicle group, and both RAGEmAb (1:10^4^ *v*/*v* diluted) and SB203580 (10 μM) respectively, caused a marked decrease of cell invasiveness versus S100B 5 μM group (−30% and −21% respectively).

### 2.2. S100B Induces VEGF-R2 and Inducible Nitric Oxide-Synthase (iNOS) Protein Expression Upregulation Causing Parallel Release of Pro-Angiogenic VEGF and NO by Cultured Caco-2 Cells

**Figure 2:**
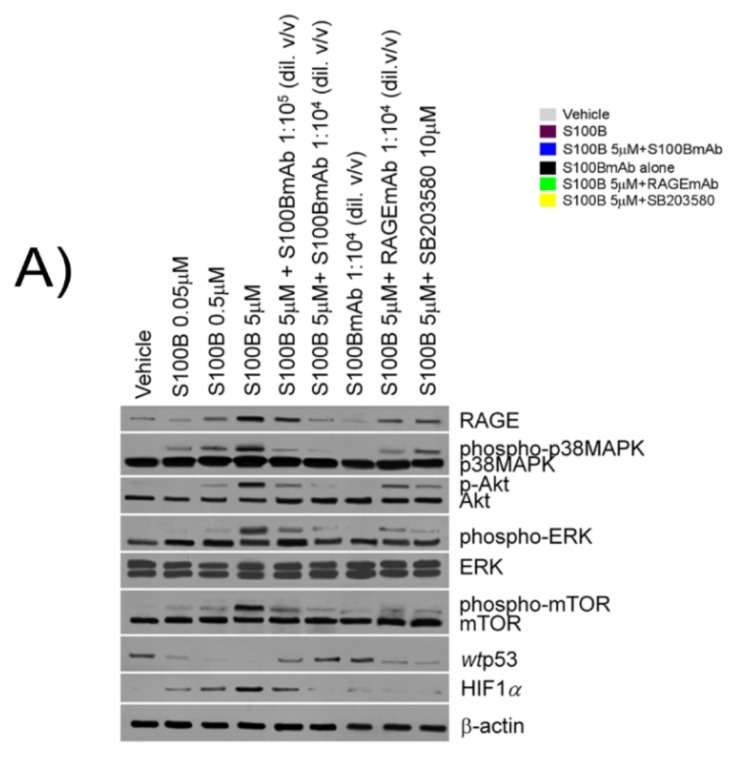

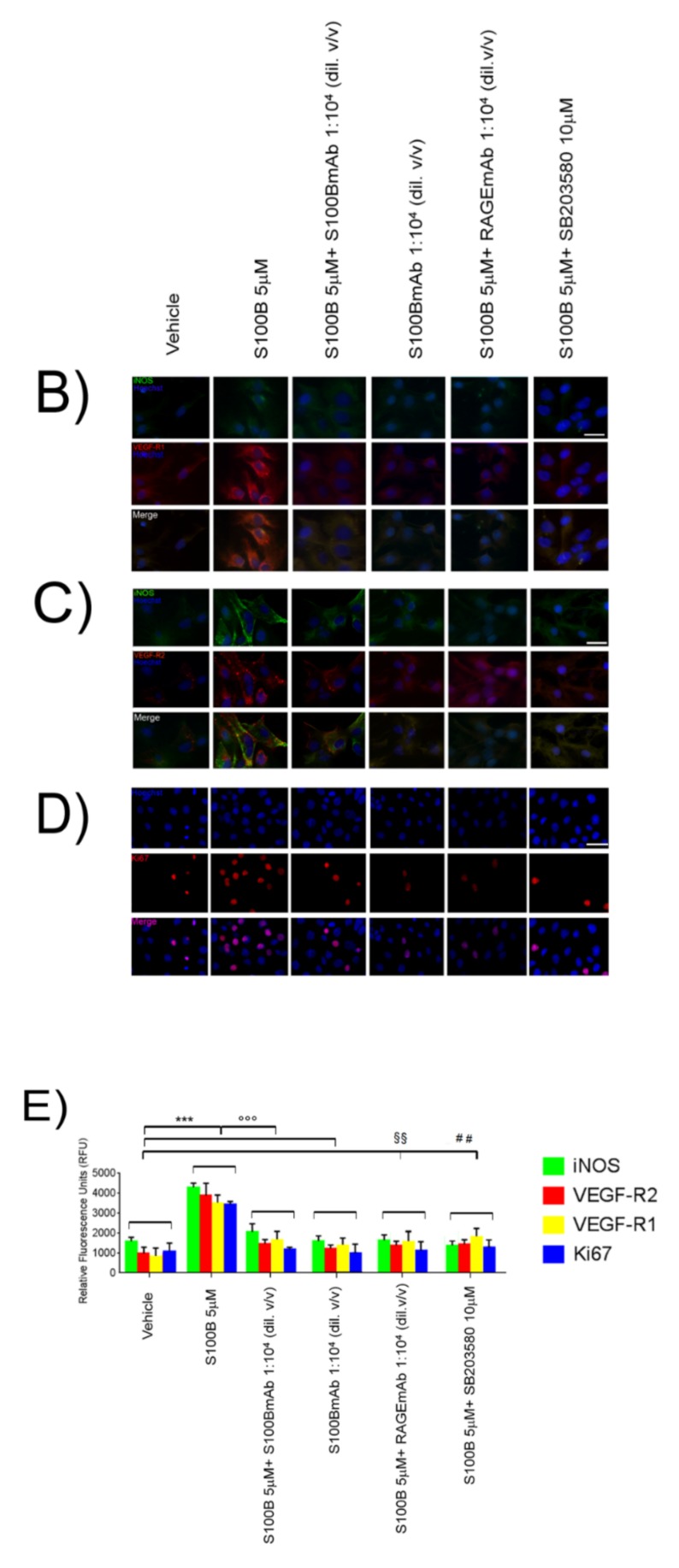

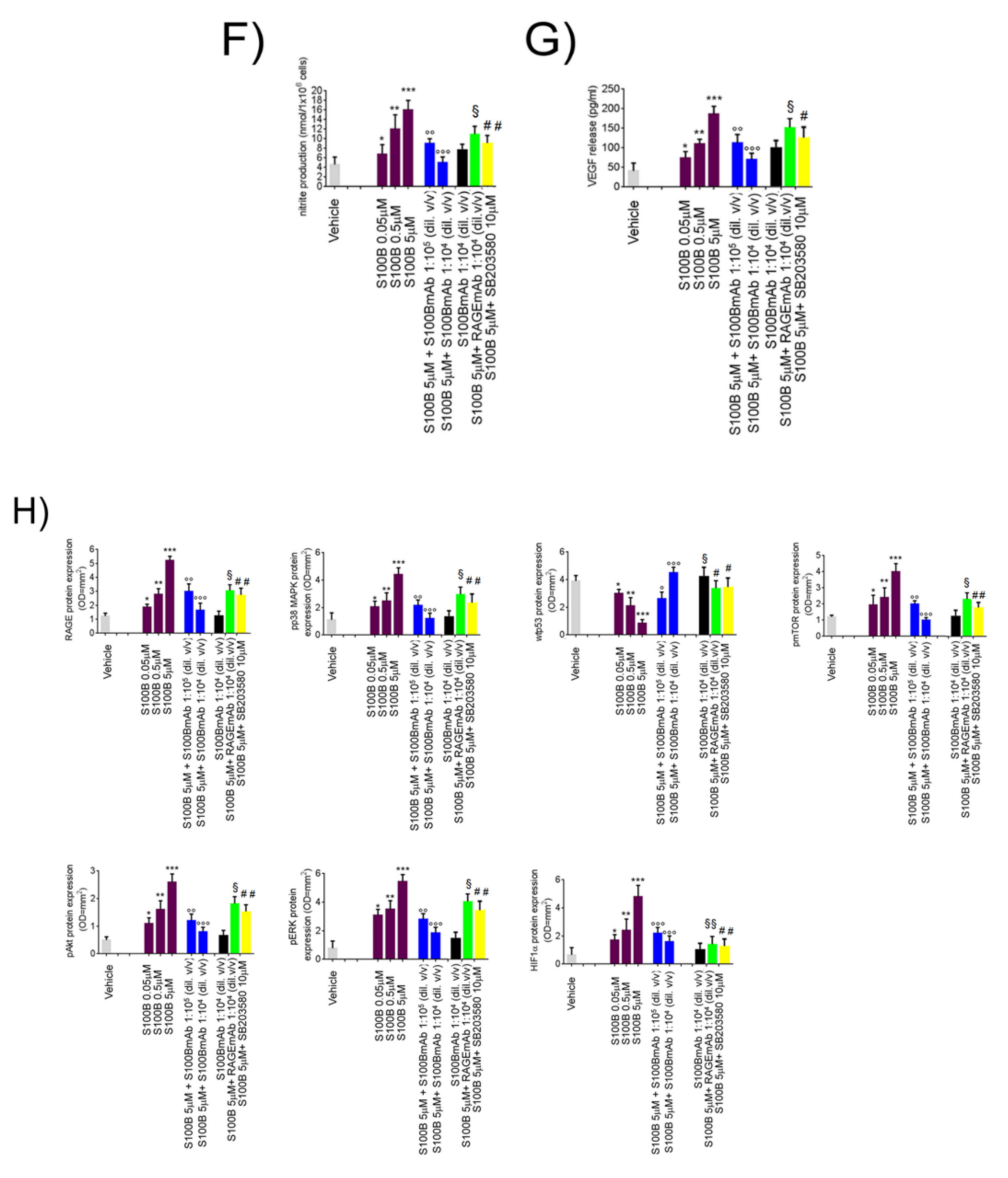
S100B stimulates pro-angiogenic vascular endothelial growth factor (VEGF) and NO release through Akt/mammalian target of rapamycin (mTOR) pathways and relative molecular downstream regulation by S100BmAb. (**A**) Immunoreactive bands referred to RAGE, phosphorylated/unphosphorylated-p38MAPK, phosphorylated/unphosphorylated Akt, phosphorylated ERK/unphosphorylated ERK, phosphorylated/unphosphorylated-mTOR, wild type (wt)p53, hypoxia-inducible factor 1-alpha (HIF1α) protein expression and (**H**) their relative densitometric analysis (arbitrary units normalized on the expression of the housekeeping protein β-actin, showing the effect of S100B (0.05–5 µM) in the presence of S100BmAb (1:10^5^–1:10^4^ *v*/*v* diluted) at 24 h, S100BmAb alone (1:10^4^ *v*/*v* diluted) or S100B 5 μM in the presence of, respectively RAGEmAb (1:10^4^ *v*/*v* diluted) or SB203580 (10 μM). (**B**) Immunofluorescence analysis showing inducible nitric oxide-synthase (iNOS; green), VEGF-R1 and (**C**) VEGF-R2 (red) and (**D**) Ki67 (red) immunoreactivity with (**E**) the relative quantification of iNOS (green bars), VEGF-R1 (yellow bars) VEGF-R2 (red bars) and Ki67 (blue bars) protein expression in Caco-2 cells treated with S100B (5 µM) in presence or absence of S100BmAb (1:10^4^ *v*/*v* diluted), or RAGEmAb (1:10^4^ *v*/*v* diluted) and SB203580 (10 μM). In the same experimental conditions, (**F** and **G**) quantification of nitrite and VEGF levels respectively in the supernatant media of Caco-2 cells exposed to the same treatment. Results were expressed as mean ± SEM of *n* = 6 experiments performed in triplicate.* *p* < 0.05; ** *p* < 0.01 and *** *p* < 0.001 versus vehicle; ° *p* < 0.05, °° *p* < 0.01 and °°° *p* < 0.001 versus S100B 5 μM; # *p* < 0.05 ## *p* < 0.01; ^§^ *p* < 0.05 and ^§§^ *p* < 0.01, respectively versus S100B 5 μM-treated cells. Scale bar: 10 µm; Magnification 10X.

Following S100B 5 μM exposure, immunofluorescence analysis revealed that both VEGF-R1 and VEGF-R2 protein were significantly increased (+250% and +268% respectively) in comparison with the vehicle group (Figure 2A–C) at 24 h. In the same experimental conditions, we also observed a significant upregulation of nuclear Ki67 protein, a marker of colonic tumor cell proliferation, as compared to control group (+221%) (Figure 2B,C). S100BmAb (1:10^4^ *v*/*v* diluted) caused a significant decrease of both VEGF-R1 (−58%), VEGF-R2 (−63%) and Ki67 (−69%), vs. S100B 5 μM group (Figure 2A–C). Our data also showed that S100B 5 μM caused a significant increase (+113%) of iNOS protein expression in comparison to vehicle group cells (Figure 2A–C) and, once again, incubation with S100BmAb (1:10^4^ *v*/*v* diluted) resulted in a concentration-dependent inhibition of iNOS protein expression (−50%), versus S100B 5 μM (Figure 2A–C).

S100BmAb (1:10^4^ *v*/*v* diluted) alone did not increase VEGF-R1, VEGF-R2, iNOS and Ki67 expression in comparison with the vehicle group, while a significant decrease of all these proteins expression was observed after coincubation with RAGEmAb (1:10^4^ *v*/*v* diluted) or SB203580 (10 μM).

Immunofluorescence analysis revealed that S100B (0.05–5 μM) caused a significant and concentration-dependent increase in both NO (expressed as nitrites amount) (+30%, +161%, +291%) and VEGF (+32%, +168% and +361%) (Figure 2F,G) released by cultured Caco-2 cells. As expected, S100BmAb (1:10^5^–1:10^4^ *v*/*v* diluted) caused both nitrite (−50% and −72%) and VEGF (−40% and −62%) (Figure 2D,E) decrease in comparison to S100B 5 μM group, while no effects on both nitrite and VEGF secretion were observed following the incubation of S100BmAb (1:10^4^ *v*/*v* diluted) alone. On the contrary, in the presence of both RAGEmAb (1:10^4^ *v*/*v* diluted) and SB203580 (10 μM), the effect of S100B 5 μM was consistently inhibited on both nitrites (−35% and −49% respectively) and VEGF secretion (−26% and −35% respectively), further supporting the role of RAGE/p38/Akt pathway in mediating the effects of S100B on pro-angiogenic factors. 

### 2.3. S100B Mediates a RAGE-Dependent Activation of p38/pAkt/pmTOR Signaling and Reduces wtp53 Expression in Cultured Caco-2 Cells

S100B (0.05–5 μM) challenge determined a significant increase of RAGE expression in comparison with the vehicle group (+30%, +128% and +298% respectively); similarly the expression of phosphorylated p38MAPK (+23%, +46% and +228%), phosphorylated-Akt (+120%, +220% and +420%), phosphorylated ERK (+260%; +320% and +470%) and phosphorylated-mTOR (+33%, +100% and +233%) was also significantly increased (Figure 2F,G). Our results showed that S100B/RAGE activation were paralleled with a downstream upregulation of hypoxia-inducible factor 1-alpha (HIF1α) (+40%, +140% and +380% vs. vehicle group respectively) (Figure 2F,G). In the same experimental conditions, S100B caused a concentration-dependent downregulation of *wt*p53 vs. the vehicle group (−23%, −46% and −79%, respectively) (Figure 2F,G). Interestingly, S100B effects were markedly counteracted by S100BmAb (1:10^5^–1:10^4^ *v*/*v* diluted) incubation. In particular, we observed a concentration-dependent decrease of RAGE (−39% and −66%), pp38 (−53% and −71%), phospho-ERK (−50% and −75%) and pAkt/pmTOR expression (−54%, −69% and −50%, −75%), respectively vs. S100B 5 μM group and HIF1α (−54% and −67% vs. S100B 5 μM group). This resulted in a significant increase of *wt*p53 protein expression (+187% and +415% respectively vs. S100B 5 µM group). In all treatment groups, S100BmAb alone did not result in any significant variation vs. vehicle group, while both RAGEmAb (1:10^4^ *v*/*v* diluted) and SB203580 were able to significantly counteract all the above-reported effects in comparison to S100B 5 μM stimulus.

## 3. Discussion

A marked release of pro-angiogenic mediators is related to poor prognosis in cancer patients [23]. The identification of molecular pathways bridging chronic inflammation to a pro-angiogenic TME setup is crucial in identifying new potential therapies for colon cancer. Previous studies showed that the release of S100B protein, a neurotrophic protein expressed by enteric glia cells in the gut, is commonly upregulated in various models of cancer and is often associated with tumor progression and prognosis [22,24,25]. Here, we demonstrated that increasing concentrations of S100B protein are accompanied by a marked increase in proliferation, migration and invasion rates in an in vitro model of human colon cancer.

Moreover, S100B challenge also had a direct pro-angiogenic effect in vitro on HUVEC cells, causing a significant increase of tube formation and length. This effect was, furthermore, significantly counteracted by the coincubation with specific S100BmAb, or RAGE blocking antibody and by the p38MAPK/Akt inhibitor, SB203580 (Figure S1).

**Figure 3:**
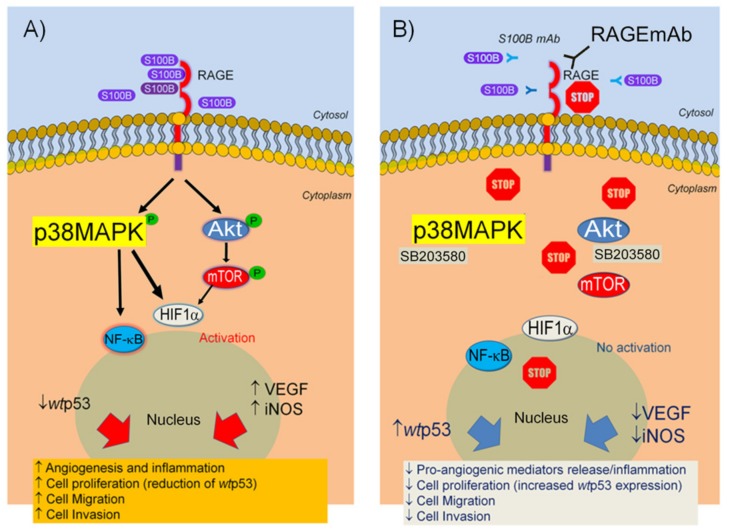
Representation of S100B effects on Caco-2 cells. (**A**) S100B interacts at RAGE receptors and downstream activates p38/Akt signaling pathway leading to cell proliferation, increased release of cell migration and angiogenesis. (**B**) S100B effects are blocked by specific S100BmAb, as well as, RAGE specific antibody and p38/Akt inhibitor SB203580, leading to a significant control of angiogenesis and cell invasion in vitro.

S100B activity led to a significant increase of both VEGF and NO release from cultured cells, that paralleled a marked upregulation of VEGF receptors 1/2 and iNOS protein expression in the same experimental conditions. VEGF and NO are crucial components of the angiogenesis network since they synergistically promote a pro-angiogenic milieu in the colon [26,27]. Different evidence produced by our research group has been addressed in the attempt to clarify the role exerted by an increased S100B level and perpetuation of chronic inflammation in the gut mucosa. In particularly, the connection between NO and S100B has been explored and the S100B/RAGE/phospho-p38MAPK interaction has been identified as a key signaling pathway by which S100B may stimulate a pro-inflammatory effect in colon mucosa [14,28,29,30]. The importance of the RAGE receptor and the relative downstream activation of p38/Akt pathway induced by exogenous S100B was supported by the observation that its proliferative and pro-angiogenic effects were significantly counteracted by either RAGEmAb or SB203580 (a synthetic p38/Akt inhibitor). In addition to this molecular pathway, here we demonstrated that S100B can also activate the pAkt-pERK/pmTOR/HIF1α signaling pathway (Figure 3), cross-linking the inflammatory cascade to neoangiogenesis and upregulation of VEGF and its receptors. These data are in line with a previous report that indicated that micromolar concentrations of S100B interact at the RAGE site and profoundly alter cell cycle and proliferation in neuronal cells, inducing pro-mitogenic activity of pAkt [20]. Notably, the activation of the Akt/mTOR pathway is downstream associated with signals promoting resistance to apoptosis, altered tissue remodeling and metastasis formation [31]. The molecular interaction between p38MAPK and pAkt/pmTOR pathway have been independently observed both in diabetic fibroblasts [32] and HeLa cells [33]. Our data are in line with previously published studies showing the mitogenic effect of S100B proteins family [34,35]. Along this line, Jiang et al [36] firstly described the effect of S100B protein on proliferation and invasion on human lung adenocarcinoma cell line PC14/B. S100B protein in particular, has been reported to sequestrate pro-apoptotic *wt*p53 protein, favoring tumor progression in several human cancers, i.e., melanoma, breast cancer or glioblastoma [17,18,37]. In a previous study from our group, S100B release was strongly increased in chronic gut inflammation, and this might, in turn, offer mechanistic insights into the transition from chronic inflammatory conditions, such as ulcerative colitis (UC) to colorectal cancer [38]. To the best of our knowledge this is the first study demonstrating that S100B has a direct-pro-angiogenic effect acting in vitro as a direct neovascularization promoter by the activation of a RAGE-dependent pAkt/mTOR pathway in a human colon adenocarcinoma cell line. We also demonstrated that the specific monoclonal anti-S100B antibody was able to counteract the above-described pro-angiogenic and proliferative effects in a concentration-dependent manner. Although hampered by their in vitro nature, these results support that the selective targeting of S100B protein might represent an interesting innovative approach in colon cancer therapy. The recent introduction of VEGF blocking antibody, bevacizumab, in the treatment of colon adenocarcinoma has significantly improved progression-free survival in advanced colon cancer patients [39]. However, the complexity and redundancy of converging angiogenic and proliferation pathways can promote escape mechanisms to immunotherapy, ultimately leading to tumor progression. The possibility, of inhibiting on one hand, the release of pro-angiogenic factors and, on the other, of promoting cell proliferation control, can be therefore of great interest in order to synergistically target these mechanisms. Here we show that monoclonal antibody-mediated targeting of S100B appears to also have a further protective anti-cancer effect by markedly increasing the expression of *wt*p53 protein.

Very interestingly, in the p53 KO HCT116 cell line, S100BmAb failed to inhibit cell proliferation rates; whilst, no significant differences were observed in terms of VEGF secretion as compared to wild type HCT116 cells and Caco-2 cell line.

Although further in vivo studies are required to confirm these in vitro preliminary results, S100B targeting might represent a promising therapeutic approach against colon cancer. Given the key role of S100B in mediating chronic inflammation and carcinogenesis, we strongly believe that the selective blockade of its effects could play an innovative and prominent role in the development of new complementary antitumor approaches that may increase therapeutic success.

## 4. Materials and Methods

### 4.1. Materials

Caco-2 cells were purchased from European Collection of Cell Cultures (ECACC, Public Health England Porton Down, Salisbury, UK). Cell medium, substances and reagents for cell cultures were all purchased from Sigma-Aldrich (St. Louis, MO, USA), unless stated otherwise. Instruments, reagents and materials for Western blot analysis were obtained from Bio-Rad Laboratories (Milan, Italy). S100B protein was from Sigma-Aldrich (St. Louis, MO, USA) and mouse monoclonal S100B antibody (S100BmAb) was purchased from AbCam (Cambridge, UK). Mouse anti-total Akt, mouse monoclonal anti p-ERK/ERK was from Santacruz biotechnology (DBA, Milan, Italy) rabbit monoclonal anti-phospho-Akt (Ser^473^), rabbit polyclonal anti-phospho-mTOR (pSer^2448^), rabbit monoclonal anti VEGF-R1 and VEGF R-2 were purchased from Cell Technology (Euroclone, Pero, Milan, Italy). Mouse monoclonal anti RAGE antibody was from AbCam (Cambridge, UK). Rabbit polyclonal anti-total mTOR was from Abcam (Cambridge, UK); mouse monoclonal anti-HIF1α was purchased from Sigma Aldrich (Milan, Italy); anti-β-actin was from Santa Cruz Biotechnology (Santa Cruz, California, USA). SB203580 was from InvivoGen (Aurogene, Rome, Italy) and polyclonal rabbit anti-mouse IgG from Dako (Glostrup, Denmark).

### 4.2. Cell Culture

Caco-2 cells obtained from ECACC (Public Health England Porton Down, Salisbury, UK) were cultured in 6-well plates in DMEM containing 10% fetal bovine serum (FBS), 1% penicillin–streptomycin, 2 mM l-glutamate and 1% non-essential amino acids to confluence. A total of 1 × 10^6^ cells/well were plated and incubated for 24 h. After reaching confluence, the cells were washed three times with phosphate-buffered saline (PBS), detached with trypsin/EDTA, plated in 10 cm diameter petri dish and allowed to adhere for 24 h. Subsequently, DMEM was replaced with fresh medium, and the cells were treated with increasing concentrations of S100B (0.05, 0.5 and 5 μM) dissolved in ultrapure and pyrogen-free sterile vehicle in the presence or absence of S100BmAb (1:10^5^–1:10^4^ *v***/***v* diluted), respectively. In the same experimental protocol, cells were also treated with S100BmAb (1:10^4^ *v*/*v* diluted) alone as an internal control group and S100B 5 μM effect was also evaluated, respectively, in the presence of RAGEmAb (1:10^4^ *v*/*v* diluted) or SB203580 (10 μM), both with p38/pAkt synthetic inhibitor. S100B and relative S100BmAb or RAGEmAb dilutions, as well as SB203580 concentration, were selected according to previously study [30,40], and pilot experiments aimed at identifying the lowest effective concentration (data not shown).

### 4.3. Western Blot

Caco-2 cells protein expression was evaluated by performing a Western blot analysis. Following treatments, cells (1 × 10^6^) were harvested, washed twice with ice-cold PBS and centrifuged at 180× *g* for 10 min at 4 °C. The cell pellet was suspended in 100 μL ice-cold hypotonic lysis buffer (10 mM 4-(2-hydroxyethyl) piperazine-1-ethanesulfonic acid, *N*-(2-hydroxyethyl) piperazine-*N*′-(2 ethanesulfonic acid) (HEPES), 1.5 mM MgCl_2_, 10 mM KCl, 0.5 mM phenylmethylsulphonylfluoride, 1.5 µg/mL soybean trypsin inhibitor, 7 µg/mL pepstatin A, 5 µg/mL leupeptin, 0.1 mM benzamidine and 0.5 mM dithiothreitol). To lyse the cells, the suspension was rapidly passed through a syringe needle five to six times prior to centrifugation for 15min at 13,000× *g* to obtain the cytoplasmic fraction. The cytosolic fraction proteins were mixed with non-reducing gel loading buffer (50 mM Tris, 10% SDS, 10% glycerol, 2 mg bromophenol/mL) at a 1:1 ratio, boiled for 3 min and centrifuged at 10,000× *g* for 10min. The protein concentration was determined using a Bradford assay, and equivalent quantities (50 µg) of each sample were electrophoresed on a 12% discontinuous polyacrylamide minigel. Subsequently, the proteins were transferred onto nitrocellulose membranes that had been saturated by incubation with 2.5% non-fat dry milk in 1× PBS overnight at 4 °C with the following antibodies: total Akt, phosphor-Akt, phosphor-ERK/total ERK, mTOR, phosphor-mTOR and anti-HIF1α, and β-actin protein expression was performed on total protein fractions of homogenates. Equivalent amounts (50 µg) of each homogenate underwent electrophoresis through a polyacrylamide minigel. Proteins were then transferred onto nitrocellulose membrane that were saturated by incubation with 10% non-fat dry milk in 1× PBS overnight at 4 °C and then incubated, according the experimental protocols with mouse anti-total Akt (1:1000 *v*/*v*, Cell Technology, Euroclone, Pero, MI, Italy), rabbit monoclonal anti-phospho-Akt (Ser^473^) (1:2000 *v*/*v*, Cell Technology, Euroclone, Pero, MI, Italy), monoclonal phosphor-ERK (1:500 *v*/*v*), rabbit polyclonal anti-total mTOR (1:1000 Abcam, Cambridge, UK), rabbit polyclonal anti-phospho-mTOR (pSer^2448^) (1:1000 *v*/*v*, Cell Technology, Euroclone, Pero, MI, Italy), mouse monoclonal anti-HIF1α (1:500 *v*/*v*, Sigma Aldrich, MI, Italy) and mouse anti-β-actin (1:2000 *v*/*v*). Membranes were then incubated with the specific secondary antibodies conjugated to horseradish peroxidase (Dako, Milan, Italy). Immune complexes were revealed by enhanced chemiluminescence detection reagents (Amersham Biosciences, Milan, Italy). Blots were analyzed by scanning densitometry (GS-700 imaging densitometer; Bio-Rad). Results were expressed as optical density (arbitrary units; mm^2^) and normalized on the expression of the housekeeping protein β-actin.

### 4.4. Enzyme-Linked Immunosorbent Assay for VEGF

Enzyme-linked immunosorbent assay for human VEGF (Abcam, Cambridge, UK) was carried out with Caco-2 cell supernatant after treatments at 24 h according to the manufacturer’s protocol. Absorbance was measured on a microtiter plate reader. VEGF levels were thus determined using the standard curves method.

### 4.5. Nitric Oxide Quantification

NO was measured as nitrite (NO_2_^−^) accumulation in the homogenates derived from Caco-2 cells treated with S100B (0.05–5 μM) in the presence or absence of S100BmAb (1:10^5^–1:10^4^ *v*/*v* diluted), or S100BmAb (1:10^4^ *v*/*v* diluted) alone, or S100B 5 μM in the presence of RAGEmAb (1:10^4^ *v*/*v* diluted) or SB203580 (10 μM)by a spectrophotometric assay based on the Reference [39] Griess reaction. Griess reagent (1 % sulphanilamide in H_2_O plus 0.1% naphthylethylenediamine in H_3_PO_4_) was added to an equal volume of supernatant and the absorbance was measured at 550 nm. The NO_2_^−^ concentration was thus determined using a standard curve of sodium nitrite and referred to 1 × 10^6^ cells.

### 4.6. Cell Proliferation Assay

Cell proliferation was evaluated by performing a 3-[4,5-dimethylthiazol-2-yl]-2,5-diphenyltetrazolium bromide (MTT) assay [41]. In brief, Caco-2 cells (5 × 10^4^) were plated in 96-well plates and allowed to adhere for 24 h. After that, DMEM was replaced with fresh medium, and the cells were treated with increasing concentrations of S100B 0.005–5 μM) dissolved in ultrapure and pyrogen-free sterile vehicle in the presence or absence of S100BmAb (1:10^5^–1:10^4^ *v*/*v* diluted) or S100BmAb (1:10^4^ *v*/*v* diluted) alone, or S100B 5 μM in the presence of RAGEmAb (1:10^4^ *v*/*v* diluted) or SB203580 (10 μM). After 48 h, 25 μL MTT (5 mg/mL MTT in DMEM) was added to the cells, and the mixture was incubated for an additional 3 h at 37 °C. Subsequently, the cells were lysed, and the dark blue crystals were solubilized using a 125 μL solution containing 50% *N*,*N*-dimethylformamide and 20% (*w*/*v*) sodium dodecylsulfate (pH 4.5). The OD of each well was determined using a PerkinElmer, Inc. (Waltham, MA, USA) microplate spectrophotometer equipped with a 620-nm filter. Cell proliferation in response to treatments was calculated using the following equation: Cell proliferation at 48 h (%) = (OD treated / OD untreated)×100.

### 4.7. Immunofluorescence Analysis

After the incubation with S100B (5 μM) in the presence or absence of S100BmAb (1:10^5^–1:10^4^ *v*/*v* diluted), or in the presence of RAGEmAb (1:10^4^ *v*/*v* diluted) or SB203580 (10 μM) or in the presence of S100BmAb alone, Caco-2 cells were harvested, fixed for 30 min in 4% paraformaldehyde, washed with ice-cold PBS and permeabilized with 0.3% Triton-X100 in PBS for 1 hour. Then, 2% bovine serum albumin (BSA) was used to block the non-specific binding sites for 2 h. The cells were then incubated overnight with mouse anti-VEGF-R1 (1:300 *v*/*v*) or anti-VEGF-R2 (1:200 *v*/*v*), rabbit anti-iNOS antibody (1:100 *v*/*v*) and mouse anti Ki67 (1:100 *v*/*v*) (all Abcam, Cambridge, UK) and further incubated in the dark for 30 min with the proper secondary antibody (fluorescein isothiocyanate (FITC)-conjugated anti-rabbit or Texas red conjugated anti-mouse; all 1:1000 *v*/*v*, Jackson Immuno-Research, Cambridge, UK). Nuclei were stained with Hoechst (1:50000 *v*/*v*, Thermo Fischer Scientific Inc., Waltham, MA, US). The cells were analyzed using a microscope (Nikon Eclipse 80i, Nikon corporation, Minato, Tokyo, Japan) and images were captured by a high-resolution digital camera (Nikon Digital Sight DS-U1, Nikon corporation, Minato, Tokyo, Japan).

### 4.8. Wound Healing Assay

A wound healing assay using the Caco-2 cells was performed as described previously, with a number of adjustments [42]. Briefly, the cells (5 × 10^5^ cells/well) were plated on a 6-well plate and incubated for 24 h in DMEM supplemented with 5% FBS, 2 mM glutamine, 100 U/mL penicillin and 100 µg/mL streptomycin in a humidified atmosphere of 5% CO_2_ and 95% air at a temperature of 37 °C. The cell layer was scratched using a 200 μL sterile pipette tip; then, cells were washed with PBS three times and untreated or incubated with S100B (0.05–5 μM) dissolved in ultrapure and pyrogen-free sterile vehicle in the presence or absence of S100BmAb (1:10^5^–1:10^4^ *v*/*v* diluted) or S100BmAb (1:10^4^ *v*/*v* diluted) alone, or S100B 5 μM in the presence of RAGEmAb (1:10^4^ *v*/*v* diluted) or SB203580 (10 μM). Caco-2 cells were then washed twice with PBS and fixed with 4% paraformaldehyde for 30 min. In order to facilitate cell counting, the nucleus of the Caco-2 cells was stained with Hoechst 33,258 (Invitrogen Life Technologies, Carlsbad, CA, USA) for 5 min at RT. The cells were subsequently washed three times with PBS, and images were captured using a Nikon Eclipse 80 microscope equipped with a high-resolution digital camera (Nikon Digital Sight DS-U1; Nikon Instruments, Inc.). The percentage of migration was calculated by counting the number of cells that had migrated into scratched areas compared with the number of cells that had remained in the peripheral areas.

### 4.9. Cell Invasion Assay

The cell invasion assay was assessed using the Millipore 24-well Millicell Chamber of pore size 8 mm (Millipore, Burlington, MA, USA), according to Zhao et al. [43]. Briefly, the cells (2 × 10^5^ cells) cells in DMEM without FBS were added into the upper chamber pre-coated with matrigel (Sigma). DMEM supplemented with 30% FBS was in the lower chamber. After the incubation with S100B (0.05–5 μM) in the presence or absence of S100BmAb (1:10^5^–1:10^4^ *v*/*v* diluted) or S100BmAb (1:10^4^ *v*/*v* diluted) alone, or S100B 5 μM in the presence of RAGEmAb (1:10^4^ *v*/*v* diluted) or SB203580 (10 μM) for 24 h, the chamber was fixed and stained with 0.5% crystal violet for 30 min and the non-invading cells were removed with cotton swabs. The number of invasive cells on the lower surface of the chamber membrane was then counted under a microscope at a magnification of 20× in five random fields.

### 4.10. Statistical Analysis

Results are expressed as the mean ± standard error (SEM) of the mean of *n* experiments. Statistical analyses were performed using one-way analysis of variance, and multiple comparisons were performed using a Bonferroni post hoc test. *p* < 0.05 was considered to indicate a statistically significant difference.

## Supplementary Materials

Supplementary Materials can be found at https://www.mdpi.com/1422-0067/20/13/3240/s1.

## Abbreviations

CRC	colorectal carcinoma
TME	tumor micro-environment
iNOS	inducible nitric oxide-synthase
VEGF	vascular endothelial growth factor
NF-κB	nuclear factor-kappaB
*wt*p53	wild type p53
S100BmAb	S100B monoclonal antibody
HEPES	4-(2-hydroxyethyl) piperazine-1-ethanesulfonic acid, *N*-(2-hydroxyethyl) piperazine-*N*′-(2 ethanesulfonic acid)
HIF1α	hypoxia-inducible factor 1-alpha
mTOR	mammalian Target of Rapamycin
RAGE	receptor for advanced glycation end products
RAGEmAb	RAGE monoclonal antibody
BSA	bovine serum albumin
MDPI	Multidisciplinary Digital Publishing Institute
DOAJ	Directory of open access journals
TLA	three letter acronym
LD	linear dichroism

## References

[1] Lipkin M. (1979). Dietary, environmental, and hereditary factors in the development of colorectal cancer. CA Cancer J. Clin..

[2] Raskov H., Pommergaard H.C., Burcharth J., Rosenberg J. (2014). Colorectal carcinogenesis—Update and perspectives. World J. Gastroenterol..

[3] Bernstein E., Caudy A.A., Hammond S.M., Hannon G.J. (2001). Role for a bidentate ribonuclease in the initiation step of RNA interference. Nature.

[4] Eaden J.A., Abrams K.R., Mayberry J.F. (2001). The risk of colorectal cancer in ulcerative colitis: A meta-analysis. Gut.

[5] Rodriguez-Caso L., Reyes-Palomares A., Sanchez-Jimenez F., Quesada A.R., Medina M.A. (2012). What is known on angiogenesis-related rare diseases? A systematic review of literature. J. Cell. Mol. Med..

[6] Catalano V., Turdo A., Di Franco S., Dieli F., Todaro M., Stassi G. (2013). Tumor and its microenvironment: a synergistic interplay. Semin. Cancer Biol..

[7] Bhattacharya R., Fan F., Wang R., Ye X., Xia L., Boulbes D., Ellis L.M. (2017). Intracrine VEGF signalling mediates colorectal cancer cell migration and invasion. Br. J. Cancer.

[8] Carmeliet P., Jain R.K. (2000). Angiogenesis in cancer and other diseases. Nature.

[9] McMahon G. (2000). VEGF receptor signaling in tumor angiogenesis. Oncologist.

[10] Bockelman C., Engelmann B.E., Kaprio T., Hansen T.F., Glimelius B. (2015). Risk of recurrence in patients with colon cancer stage II and III: A systematic review and meta-analysis of recent literature. Acta Oncol..

[11] Cantley L.C. (2002). The phosphoinositide 3-kinase pathway. Science.

[12] Vivanco I., Sawyers C.L. (2002). The phosphatidylinositol 3-Kinase AKT pathway in human cancer. Nat. Rev. Cancer.

[13] Li W., Tan D., Zhang Z., Liang J.J., Brown R.E. (2008). Activation of Akt-mTOR-p70S6K pathway in angiogenesis in hepatocellular carcinoma. Oncol. Rep..

[14] Cirillo C., Sarnelli G., Esposito G., Turco F., Steardo L., Cuomo R. (2011). S100B protein in the gut: the evidence for enteroglial-sustained intestinal inflammation. World J. Gastroenterol..

[15] Moravkova P., Kohoutova D., Rejchrt S., Cyrany J., Bures J. (2016). Role of S100 Proteins in Colorectal Carcinogenesis. Gastroenterol. Res. Pract..

[16] Hartman K.G., McKnight L.E., Liriano M.A., Weber D.J. (2013). The evolution of S100B inhibitors for the treatment of malignant melanoma. Future Med. Chem..

[17] Wang H., Zhang L., Zhang I.Y., Chen X., Da Fonseca A., Wu S., Ren H., Badie S., Sadeghi S., Ouyang M. (2013). S100B promotes glioma growth through chemoattraction of myeloid-derived macrophages. Clin. Cancer Res..

[18] Holla F.K., Postma T.J., Blankenstein M.A., van Mierlo T.J.M., Vos M.J., Sizoo E.M., de Groot M., Uitdehaag B.M.J., Buter J., Klein M. (2016). Prognostic value of the S100B protein in newly diagnosed and recurrent glioma patients: a serial analysis. J. Neurooncol..

[19] Donato R., Cannon B.R., Sorci G., Riuzzi F., Hsu K., Weber D.J., Geczy C.L. (2013). Functions of S100 proteins. Curr. Mol. Med..

[20] Leclerc E., Fritz G., Vetter S.W., Heizmann C.W. (2009). Binding of S100 proteins to RAGE: an update. Biochim. Biophys. Acta.

[21] Chen X., Zhang L., Zhang I.Y., Liang J., Wang H., Ouyang M., Wu S., da Fonseca A.C.C., Weng L., Yamamoto Y. (2014). RAGE expression in tumor-associated macrophages promotes angiogenesis in glioma. Cancer Res..

[22] Lin J., Yang Q., Yan Z., Markowitz J., Wilder P.T., Carrier F., Weber D.J. (2004). Inhibiting S100B restores p53 levels in primary malignant melanoma cancer cells. J. Biol. Chem..

[23] Liu Z., Qi L., Li Y., Zhao X., Sun B. (2017). VEGFR2 regulates endothelial differentiation of colon cancer cells. BMC Cancer.

[24] Andres R., Mayordomo J.I., Zaballos P., Rodino J., Isla D., Escudero P., Elosegui L., Filipovich E., Saenz A., Polo E. (2004). Prognostic value of serum S-100B in malignant melanoma. Tumori.

[25] Capoccia E., Cirillo C., Gigli S., Pesce M., D’Alessandro A., Cuomo R., Sarnelli G., Steardo L., Esposito G. (2015). Enteric glia: A new player in inflammatory bowel diseases. Int. J. Immunopathol. Pharmacol..

[26] Ahluwalia A., Jones M.K., Matysiak-Budnik T., Tarnawski A.S. (2014). VEGF and colon cancer growth beyond angiogenesis: does VEGF directly mediate colon cancer growth via a non-angiogenic mechanism?. Curr. Pharm. Des..

[27] Gao Y., Zhou S., Xu Y., Sheng S., Qian S.Y., Huo X. (2019). Nitric oxide synthase inhibitors 1400W and L-NIO inhibit angiogenesis pathway of colorectal cancer. Nitric Oxide.

[28] Esposito G., Capoccia E., Turco F., Palumbo I., Lu J., Steardo A., Cuomo R., Sarnelli G., Steardo L. (2014). Palmitoylethanolamide improves colon inflammation through an enteric glia/toll like receptor 4-dependent PPAR-alpha activation. Gut.

[29] Cirillo C., Sarnelli G., Esposito G., Grosso M., Petruzzelli R., Izzo P., Cali G., D’Armiento F.P., Rocco A., Nardone G. (2009). Increased mucosal nitric oxide production in ulcerative colitis is mediated in part by the enteroglial-derived S100B protein. Neurogastroenterol. Motil..

[30] Esposito G., Cirillo C., Sarnelli G., De Filippis D., D’Armiento F.P., Rocco A., Nardone G., Petruzzelli R., Grosso M., Izzo P. (2007). Enteric glial-derived S100B protein stimulates nitric oxide production in celiac disease. Gastroenterology.

[31] You S., Li W., Guan Y. (2018). Tunicamycin inhibits colon carcinoma growth and aggressiveness via modulation of the ERK-JNK-mediated AKT/mTOR signaling pathway. Mol. Med. Rep..

[32] Nishikai-Yan Shen T., Kanazawa S., Kado M., Okada K., Luo L., Hayashi A., Mizuno H., Tanaka R. (2017). Interleukin-6 stimulates Akt and p38 MAPK phosphorylation and fibroblast migration in non-diabetic but not diabetic mice. PLoS ONE.

[33] Kim M.J., Choi S.Y., Park I.C., Hwang S.G., Kim C., Choi Y.H., Kim H., Lee K.H., Lee S.J. (2008). Opposing roles of c-Jun NH2-terminal kinase and p38 mitogen-activated protein kinase in the cellular response to ionizing radiation in human cervical cancer cells. Mol. Cancer Res..

[34] Chen H., Xu C., Jin Q., Liu Z. (2014). S100 protein family in human cancer. Am. J. Cancer Res..

[35] Bresnick A.R., Weber D.J., Zimmer D.B. (2015). S100 proteins in cancer. Nat. Rev. Cancer.

[36] Jiang W., Jia Q., Liu L., Zhao X., Tan A., Ma N., Zhang H. (2011). S100B promotes the proliferation, migration and invasion of specific brain metastatic lung adenocarcinoma cell line. Cell Biochem. Funct..

[37] Yen M.C., Huang Y.C., Kan J.Y., Kuo P.L., Hou M.F., Hsu Y.L. (2018). S100B expression in breast cancer as a predictive marker for cancer metastasis. Int. J. Oncol..

[38] Esposito G., Capoccia E., Tiberi S., D’Alessandro A., Pesce M., Steardo L., Palumbo I., Cuomo R., Sarnelli G. (2014). The S100B-P53 protein-protein interaction: A novel role for enteric glia in colon cancer. In UEG week 2014, Vienne, Austria, 18-22 October 2014. United Europ. Gastroenterol. J..

[39] Qu C.Y., Zheng Y., Zhou M., Zhang Y., Shen F., Cao J., Xu L.M. (2015). Value of bevacizumab in treatment of colorectal cancer: A meta-analysis. World J. Gastroenterol..

[40] Lali F.V., Hunt A.E., Turner S.J., Foxwell B.M. (2000). The pyridinyl imidazole inhibitor SB203580 blocks phosphoinositide-dependent protein kinase activity, protein kinase B phosphorylation, and retinoblastoma hyperphosphorylation in interleukin-2-stimulated T cells independently of p38 mitogen-activated protein kinase. J. Biol. Chem..

[41] Mosmann T. (1983). Rapid colorimetric assay for cellular growth and survival: application to proliferation and cytotoxicity assays. J. Immunol. Methods.

[42] Renault-Mihara F., Beuvon F., Iturrioz X., Canton B., De Bouard S., Leonard N., Mouhamad S., Sharif A., Ramos J.W., Junier M.P. (2006). Phosphoprotein enriched in astrocytes-15 kDa expression inhibits astrocyte migration by a protein kinase C delta-dependent mechanism. Mol. Biol. Cell..

[43] Zhao F., Li L., Guan L., Yang H., Wu C., Liu Y. (2014). Roles for GP IIb/IIIa and alphavbeta3 integrins in MDA-MB-231 cell invasion and shear flow-induced cancer cell mechanotransduction. Cancer Lett..

