# Supplementary material for: S100B Protein Stimulates Proliferation and Angiogenic Mediators Release through RAGE/pAkt/mTOR Pathway in Human Colon Adenocarcinoma Caco-2 Cells
Source: Int J Mol Sci. 2019 Jul 1;20(13):3240. doi: 10.3390/ijms20133240 (PMC6651655; doi:10.3390/ijms20133240)
Supplement: Supplementary file 1 [file ijms-20-03240-s001.pdf]

S100B Protein Stimulates Proliferation and Angiogenic Mediators Release through RAGE/pAkt/mTOR Pathway in Human Colon Adenocarcinoma Caco-2 Cells

Materials and Methods

Human Umbilical Vein Endothelial Cells Culture and Tube Formation Assay

HUVEC cells were provided by the Dr. Teresa Iuvone Department of Pharmacology (University Naples, Italy) and were cultured in Clonetics® EGM®Endothelial Growth Medium (Lonza Basel, CH) at 37 °C, 5% (*v/v*) CO_2. After this time cells were incubated for 48 h with S100B 5 μM given alone or in the presence of S100BmAb (1:10^4 *v/v* diluted). In the same experimental protocol, the effect of S100BmAb (1:10^4 *v/v* diluted) alone was also evaluated and the effect of RAGEmAb (1:10^4 *v/v* diluted) and SB203580 (10 μM) was also tested in the presence of S100B (5 μM) stimulus to test in vitro tube formation as indicative of angiogenic process according to Cultrex® In Vitro Angiogenesis Assay Tube Formation Kit instruction's protocol. Cells were imaged under optical microscopy Nikon Eclipse 80i and the number of branch points per field (mm^2) and the mean tube length (mm/field) were measured using ImageJ software.

HCT116 Cell Culture, Proliferation and VEGF Release Assay

Wild type human colon carcinoma cells, HCT116 and null p53 HCT116, were generously gifted by Dr. Teresa Iuvone (Department of Pharmacology University Naples, Italy). Cells were grown in RPMI 1640 supplemented with 10% fetal calf serum, 100 U/ml penicillin, 100 μg/ml streptomycin, and 2 mM L-glutamine. Cells were maintained at 37 °C with 5% CO_2 until reaching 85%–90% confluency. Before treatments, cells were harvested by trypsin and re-suspended in Hanks balanced salt solution (HBSS) without calcium or magnesium. Both cells type were incubated for 48 h with increasing concentrations of S100B (0.05–5 μM) in the presence of S100BmAb (1:10^5 and 1:10^4 *v/v* diluted). In the same experimental protocol, the effect of S100BmAb (1:10^4 *v/v* diluted) alone was also evaluated and the effect of RAGEmAb (1:10^4 *v/v* diluted) and SB203580 (10 μM) was also tested in the presence of S100B (5 μM) stimulus.

As previously described, cell proliferation was evaluated by performing a 3-[4,5-dimethylthiazol-2-yl]-2,5-diphenyltetrazolium bromide (MTT) assay [41]. Cell proliferation in response to treatments was calculated using the following equation: Cell proliferation at 48 h (%) = (OD treated / OD untreated) × 100. Enzyme-linked immunosorbent assay for human VEGF (Abcam, Cambridge, UK) was carried out on both HUVEC, *wt* and p53 null HCT116 cell supernatant after treatments at 24 h according to the manufacturer's protocol. Absorbance was measured on a microtiter plate reader. VEGF levels were thus determined using the standard curves method.

Results

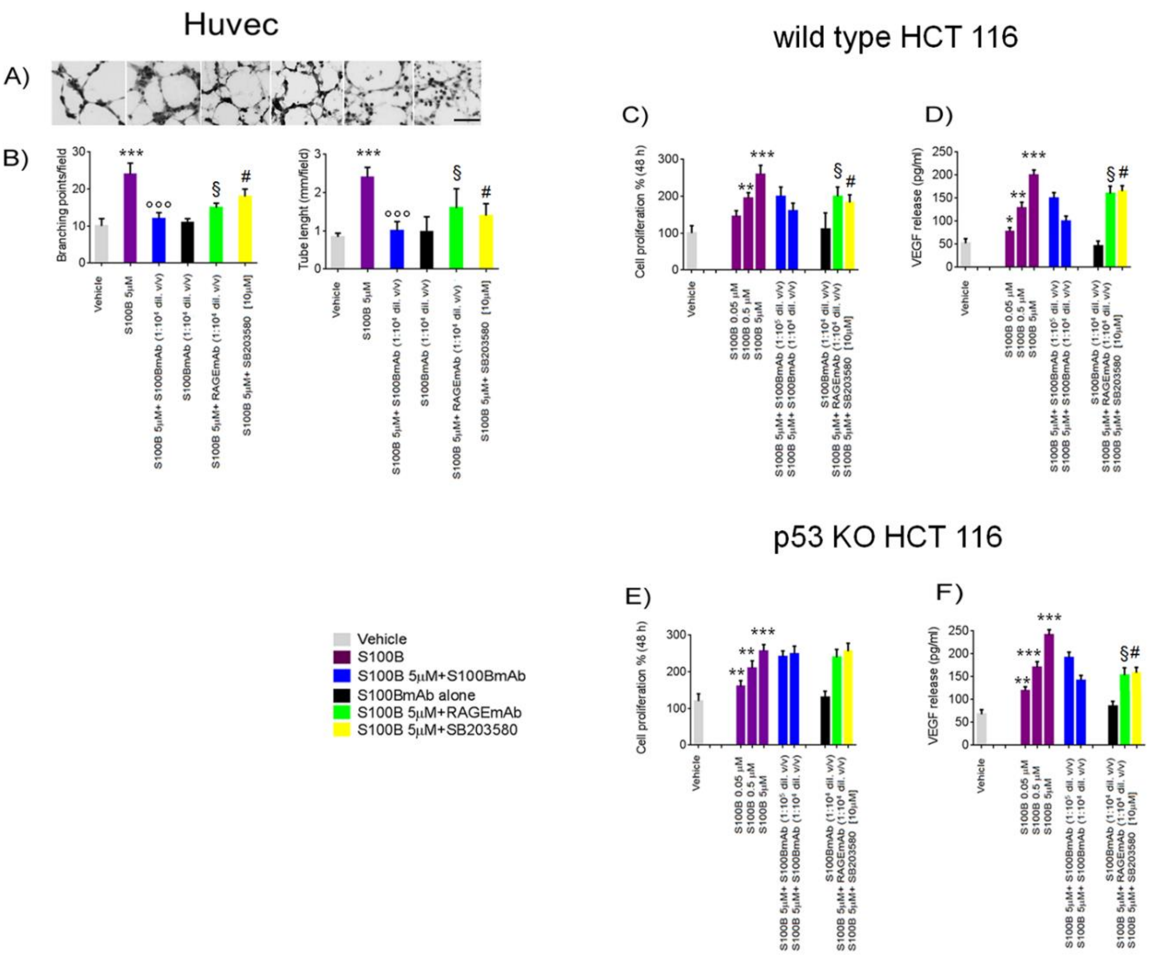

Figure S1. Exogenous S100B protein stimulates tube formation and promotes VEGF release by human umbilical vein endothelial cells **(**HUVEC). Moreover, S100B increases cell proliferation and VEGF release through Akt/mTOR pathway in wild type HCT116 cell. (**A**) Microscopy images of tube formation, relative quantification of in vitro angiogenesis by (**B**) branching points/field quantification and tube length measurement, and (**C**) effect of S100B (5 µM) on VEGF release by cultured HUVEC cells in the presence of S100BmAb (1:10^4 *v/v* diluted) , RAGEmAb (1:10^4 *v/v* diluted) or SB203580 (10 µM) treatments. Cell proliferation and VEGF release in (**D**–**G**) both wild type HCT116 and (**E**–**F**) p53KO HCT116 cells exposed to S100B (5 µM) in the presence of S100BmAb (1:10^4 *v/v* diluted), RAGEmAb (1:10^4 *v/v* diluted) or SB203580 (10 µM) treatments. Results were expressed as mean ± SEM of n = 6 experiments performed in triplicate.* $p < 0.05$; ** $p < 0.01$ and *** $p < 0.001$ versus vehicle; ° $p < 0.05$, °° $p < 0.01$ and °°° $p < 0.001$ versus S100B 5 µM; # $p < 0.05$; § $p < 0.05$, respectively versus S100B 5 µM -treated cells. Scale bar: 100 µm. Magnification10X.
